# Susceptibility of Gram-negative isolates collected in South Korea to imipenem/relebactam and comparator agents—SMART 2018–21

**DOI:** 10.1093/jacamr/dlad149

**Published:** 2023-12-28

**Authors:** James A Karlowsky, Mark G Wise, Wei-Ting Chen, Fakhar Siddiqui, Katherine Young, Mary R Motyl, Daniel F Sahm

**Affiliations:** IHMA, Schaumburg, IL, USA; Department of Medical Microbiology and Infectious Diseases, Max Rady College of Medicine, University of Manitoba, Winnipeg, Manitoba, Canada; IHMA, Schaumburg, IL, USA; MSD, Taipei, Taiwan; Merck & Co., Inc., Rahway, NJ, USA; Merck & Co., Inc., Rahway, NJ, USA; Merck & Co., Inc., Rahway, NJ, USA; IHMA, Schaumburg, IL, USA

## Abstract

**Objectives:**

To evaluate the *in vitro* susceptibility of recent Gram-negative pathogens collected in South Korean medical centres to imipenem/relebactam and comparator agents.

**Methods:**

From 2018 to 2021, six hospitals in South Korea each collected up to 250 consecutive, aerobic or facultative Gram-negative pathogens per year from patients with bloodstream, intra-abdominal, lower respiratory tract and urinary tract infections. MICs were determined using CLSI broth microdilution and interpreted by 2023 CLSI breakpoints. Most isolates that were imipenem/relebactam, imipenem or ceftolozane/tazobactam non-susceptible were screened for β-lactamase genes by PCR or WGS.

**Results:**

Of all non-Morganellaceae Enterobacterales (NME) isolates (*n *= 4100), 98.8% were imipenem/relebactam susceptible. Most NME were also susceptible to imipenem alone (94.7%) and meropenem (97.3%); percent susceptible values for non-carbapenem β-lactam comparators were lower (68%–80%). Imipenem/relebactam retained activity against 96.4%, 70.8% and 70.6% of MDR, difficult-to-treat resistant (DTR) and meropenem-non-susceptible NME, respectively, and inhibited 93.1% of KPC-carrying and 95.5% of ESBL-carrying NME. Of imipenem/relebactam-resistant NME, 21/25 (84.0%) carried an MBL or an OXA-48-like carbapenemase. Of all *Pseudomonas aeruginosa* isolates (*n *= 738), 82.8% were imipenem/relebactam susceptible; percent susceptible values for all β-lactam comparators, including carbapenems (imipenem, meropenem) were 61.5%–74.7%. Less than 20% of MDR and DTR isolates, and 41% of meropenem-non-susceptible *P. aeruginosa* isolates were imipenem/relebactam susceptible. Of imipenem/relebactam-resistant *P. aeruginosa* isolates, 61.6% carried an MBL and 37.0% did not possess any acquired β-lactamase genes.

**Conclusions:**

Based on *in vitro* data, imipenem/relebactam, if licensed in South Korea, may be a viable treatment option for many hospitalized patients infected with common Gram-negative pathogens including NME exhibiting MDR, DTR and carbapenem resistance and many β-lactam-resistant phenotypes of *P. aeruginosa*.

## Introduction

Infection caused by carbapenem-resistant Enterobacterales (CRE) and carbapenem-resistant *Pseudomonas aeruginosa* (CRPA) are a global concern. CRE and CRPA are easily transmissible pathogens that frequently demonstrate MDR and difficult-to-treat resistance (DTR) phenotypes leaving few, often toxic and therapeutically ineffective treatments, particularly for serious Gram-negative infections. Continuous monitoring of Gram-negative bacilli for resistance to β-lactams, fluoroquinolones and other potential bactericidal therapies at local, regional, national and international levels is critical to antimicrobial stewardship, infection control and drug development initiatives. Identifying and tracking changes in the underlying mechanisms of antimicrobial resistance, particularly β-lactamases, is also essential to support these initiatives.

A limited number of new β-lactam/β-lactamase inhibitor combinations, which pair an established β-lactam with a non-β-lactam β-lactamase inhibitor, have recently been licensed in some countries. One of these combinations, imipenem/relebactam, unites imipenem/cilastatin with relebactam, a bicyclic diazabicyclooctane inhibitor of class A and C β-lactamases.^1^ Imipenem/relebactam is approved by the EMA and the FDA for hospital-acquired and ventilator-associated bacterial pneumonia (HAP and VAP), bacteraemia associated with HAP and VAP (EMA only), and infections due to aerobic Gram-negative bacilli in adults with limited treatment options [e.g. complicated urinary tract infection (UTI), complicated intra-abdominal infection].^1^

In the current study, we evaluated the activity of imipenem/relebactam and relevant comparators against clinical isolates of Gram-negative bacilli collected by clinical laboratories in South Korea as part of the Study for Monitoring Antimicrobial Resistance Trends (SMART) global surveillance programme.

## Materials and methods

### Bacterial isolates

During 2018–21, six clinical laboratories in South Korea participated in the SMART global surveillance programme. Each laboratory collected consecutive, aerobic or facultative Gram-negative isolates from intra-abdominal infections (50 isolates/year), UTIs (50 isolates/year), lower respiratory tract infections (100 isolates/year) and bloodstream infections (50 isolates/year). Only one isolate per patient per species per year was accepted. All isolates were sent to a central laboratory (IHMA, Schaumburg, IL, USA), where species identity was confirmed using MALDI-TOF MS (Bruker Daltonics, Billerica, MA, USA) and antimicrobial susceptibility and molecular testing were performed. Of the 5695 Gram-negative bacilli collected during 2018–21, 4235 isolates were Enterobacterales and 738 isolates were *P. aeruginosa* (the remaining isolates were other Gram-negative species not examined for this study). Because species in the Morganellaceae family of Enterobacterales, which include the genera *Proteus*, *Providencia* and *Morganella*, often display elevated imipenem (and imipenem/relebactam)^2^ MICs by mechanisms other than the production of carbapenemases, analyses of Enterobacterales were restricted to the 4100 isolates of non-Morganellaceae Enterobacterales (NME) (96.8% of all Enterobacterales isolates collected).

### Antimicrobial susceptibility testing

MICs were determined by the CLSI reference broth microdilution method^3^ using broth microdilution panels prepared at IHMA. MICs were interpreted using 2023 CLSI breakpoints^2^ with the following exceptions. Amikacin MICs for Enterobacterales were interpreted using the EUCAST susceptible breakpoint (≤8 mg/L)^4^ because the current CLSI susceptible breakpoint (revised in 2023) is lower than the lowest concentration tested on the broth microdilution panel used in this study (MIC testing of isolates occurred from 2018 to 2022). Amikacin MICs for *P. aeruginosa* were interpreted using the CLSI urine isolate susceptible breakpoint (≤16 mg/L) (introduced in 2023) because the CLSI no longer publishes systemic MIC breakpoints for this agent tested against *P. aeruginosa*.^2^ Colistin MICs for both Enterobacterales (susceptible,  ≤ 2 mg/L) and *P. aeruginosa* (≤4 mg/L) were interpreted using the EUCAST susceptibility breakpoints^4^ because CLSI does not publish a susceptible breakpoint for this agent for any pathogen. MDR was defined as resistance to ≥3 sentinel agents [amikacin, aztreonam, cefepime, ceftazidime (Enterobacterales only), colistin, imipenem, levofloxacin and piperacillin/tazobactam]. DTR phenotypes were identified using the criteria published by Kadri *et al*.^5^ Specifically, DTR phenotypes were defined by isolates non-susceptible (intermediate or resistant) to all β-lactams (including aztreonam, ceftazidime, cefepime, imipenem, meropenem, piperacillin/tazobactam), as well as fluoroquinolones (levofloxacin).

### Screening for β-lactamase genes

Isolates meeting the following phenotypic criteria were screened for β-lactamase genes: NME isolates (excluding *Serratia* spp.) with imipenem or imipenem/relebactam MIC values of ≥2 mg/L and *P. aeruginosa* isolates with imipenem or imipenem/relebactam MIC values of ≥4 mg/L; NME and *Serratia* spp. isolates with ertapenem MIC values of ≥1 mg/L collected in 2018 only; isolates of *Serratia* spp. with imipenem MIC values of ≥4 mg/L collected in 2018; and Enterobacterales and *P. aeruginosa* isolates with ceftolozane/tazobactam MIC values of ≥4 mg/L and ≥8 mg/L, respectively. Previously published multiplex PCR assays were used to screen for the following β-lactamase genes: ESBLs (CTX-M, GES, PER, SHV, TEM, VEB); acquired AmpC β-lactamases (ACC, ACT, CMY, DHA, FOX, MIR, MOX) and the chromosomal AmpC intrinsic to *P. aeruginosa* (PDC); serine carbapenemases [GES, KPC, OXA-48-like (Enterobacterales), OXA-24-like (*P. aeruginosa*)]; and MBLs (GIM, IMP, NDM, SPM, VIM).^6,7^ All detected genes encoding carbapenemases, ESBLs and PDC were amplified using gene-flanking primers and sequenced (Sanger). For *P. aeruginosa* collected in 2020 and 2021 only, isolates were characterized by short-read WGS (Illumina HiSeq 2 × 150 bp reads) to a targeted coverage depth of 100×^8^ and analysed using the CLC Genomics Workbench (QIAGEN). The ResFinder database was used to detect β-lactamase genes in WGS assemblies.^9^ Per SMART protocol for Enterobacterales isolates collected in 2021, a representative sample of approximately 95% of isolates meeting the criteria for molecular characterization were characterized. Accordingly, of the 169 Enterobacterales isolates that met the testing criteria that year, nine randomly selected isolates were not molecularly characterized. Per SMART protocol for *P. aeruginosa* isolates collected in 2020 and 2021, a representative sample of approximately 75% of isolates meeting the criteria for molecular characterization were characterized (35 randomly selected isolates of 158 qualified isolates were not characterized).

## Results

The addition of relebactam to imipenem inhibited the growth of 98.8% (susceptible) of all NME isolates compared with 94.7% susceptible to imipenem alone (Table 1). Comparator antimicrobial agents, including meropenem (97.3% susceptible), amikacin (96.2% of MICs ≤8 mg/L) and colistin (97.5% of MICs ≤2 mg/L) were slightly less active *in vitro* than imipenem/relebactam. Imipenem/relebactam was highly active against the two most prevalent species of Enterobacterales collected, *Escherichia coli* (99.7% susceptible) and *Klebsiella pneumoniae* (97.8%), as well as against *Enterobacter* spp. (99.0%) and *Citrobacter* spp. (98.5%). Imipenem/relebactam was less active against *Serratia* spp. (89.4% susceptible) than against more commonly isolated species of Enterobacterales.

**Table 1.** Antimicrobial susceptibility of clinical isolates of Gram-negative bacilli collected in South Korea in 2018–21

	% Susceptible									
Organism/group (*n*)	IMR	IPM	MEM	ATM	FEP	CAZ	TZP	LVX^a^	AMK^b^	CST^c^
NME										
All (4100)	98.8	94.7	97.3	68.3	70.6	70.8	80.1	58.5	96.2	97.5
*E. coli* (1922)	99.7	99.3	99.6	67.0	65.6	72.4	90.7	44.8	95.7	99.9
*K. pneumoniae* (1458)	97.8	91.8	93.8	67.7	68.0	67.6	70.0	64.8	95.3	99.3
*Enterobacter* spp. (194)	99.0	90.2	98.5	58.2	75.3	55.7	59.3	77.3	99.0	84.0
*Citrobacter* spp. (133)	98.5	90.2	98.5	59.4	91.7	59.4	63.2	70.7	99.2	100
*Serratia* spp. (66)	89.4	84.8	98.5	95.5	95.5	98.5	89.4	72.7	100	16.7
MDR (990)	96.4	87.0	89.0	1.8	11.8	3.6	36.0	16.1	88.5	98.2
DTR (96)	70.8	0	0	0	0	0	0	0	90.6	93.8
MEM-NS (109)	70.6	3.7	0	1.8	0.9	2.8	0.9	6.4	90.8	94.5
TZP-NS (816)	95.6	84.1	86.8	19.2	33.3	19.6	0	27.9	88.1	97.3
FEP-NS (1205)	96.8	89.9	91.0	8.4	0	18.2	54.9	17.0	91.1	98.9
KPC-positive^d^ (72)	93.1	0	0	0	0	1.4	0	4.2	94.4	94.4
ESBL-positive^e^ (157)	95.5	88.5	95.5	3.2	3.2	2.5	7.6	5.7	82.8	94.4
*P. aeruginosa*										
All (738)	82.8	61.5	71.7	66.9	74.7	71.4	67.1	58.9	88.9	99.5
MDR (133)	17.3	2.3	4.5	32.3	8.3	14.3	3.0	1.5	43.6	97.0
DTR (74)	17.6	0	0	0	0	0	0	0	56.8	100
MEM-NS (209)	40.7	5.7	0	31.6	34.4	34.9	21.5	16.3	63.6	98.1
TZP-NS (243)	52.3	29.2	32.5	28.0	28.4	21.4	0	22.6	67.9	98.4
CAZ-NS (211)	50.2	30.3	35.5	30.8	25.6	0	9.5	28.0	65.4	98.1

IMR, imipenem/relebactam; IPM, imipenem; MEM, meropenem; ATM, aztreonam; FEP, cefepime; CAZ, ceftazidime; TZP, piperacillin/tazobactam; LVX, levofloxacin; AMK, amikacin; CST, colistin; NME, non-Morganellaceae Enterobacterales; DTR, difficult-to-treat resistance; NS, non-susceptible.

^a^LVX data exclude *Salmonella* spp. (*n *= 6) as the MIC panel dilution range did not extend into susceptible concentrations for that genus.

^b^For AMK, percent susceptible values for NME were calculated using EUCAST 2023 breakpoints^4^ as the MIC panel dilution range did not cover the CLSI susceptible breakpoint. For *P. aeruginosa*, CLSI MIC breakpoints for AMK applicable to UTI isolates were applied to all isolates.^2^

^c^For CST, EUCAST 2023 MIC breakpoints^4^ were utilized because CLSI does not publish susceptibility breakpoints for Enterobacterales or *P. aeruginosa*.

^d^Excludes isolates co-carrying MBLs.

^e^Excludes isolates co-carrying carbapenemases.

Greater percentages of MDR (96.4%), piperacillin/tazobactam-non-susceptible (95.6%) and cefepime-non-susceptible (96.8%) NME were susceptible to imipenem/relebactam than to all comparator agents except colistin. The addition of relebactam to imipenem increased the percent susceptible value for carbapenem (meropenem)-non-susceptible isolates from 3.7% (imipenem alone) to 70.6% (imipenem/relebactam).

Overall, 72 NME (1.8% of all 4100 isolates) carried KPC (not including 3 isolates co-carrying an MBL), and 93.1% of these KPC-positive isolates were imipenem/relebactam susceptible. All comparator β-lactams were inactive versus the KPC carriers. Among the 157 carbapenemase-negative, ESBL-harbouring isolates identified, imipenem/relebactam and meropenem were the most active agents, both inhibiting 95.5% of isolates.

Against the full collection of 738 *P. aeruginosa* isolates, a greater percentage were susceptible to imipenem/relebactam (82.8%) than to comparator β-lactam antimicrobials and levofloxacin (Table 1). Imipenem/relebactam was largely inactive versus *P. aeruginosa* identified as MDR or DTR, as only 17.3%–17.6% of these resistant subsets tested as imipenem/relebactam susceptible. Approximately 41% of meropenem-non-susceptible *P. aeruginosa* and 50% of ceftazidime- and piperacillin/tazobactam-non-susceptible *P. aeruginosa* were susceptible to imipenem/relebactam, greater percentages than comparator β-lactams and levofloxacin.

Table 2 summarizes the percent susceptible values for NME and *P. aeruginosa* to imipenem/relebactam and comparators stratified by isolate infection source, hospital ward type (ICU versus non-ICU wards) and length of stay [i.e. presumed nosocomially acquired infections (length of hospitalization ≥48 h) and presumed community-acquired infections (length of hospitalization <48 h)]. For NME, percent susceptible values to imipenem/relebactam were largely consistent among all collection variables, displaying a narrow range from 98% to 99%; imipenem/relebactam was the most active agent for each of the categories examined. For *P. aeruginosa*, percent susceptible values for all agents were lower for UTI isolates than for isolates from other infection sources, with the notable exception of aztreonam, for which percent susceptible values were similar for UTI isolates (63.6%) and intra-abdominal infection isolates (63.1%). Small differences were noted between the percent susceptible values for imipenem/relebactam for ICU patient isolates (80.8%) versus non-ICU patient isolates (83.3%), and isolates from patients with presumed community-acquired infections (88.5%) versus hospital-acquired infections (79.7%).

**Table 2.** Antimicrobial susceptibility of clinical isolates of Gram-negative bacilli collected in South Korea in 2018–21 stratified by infection source, hospital ward and length of hospitalization

	% Susceptible									
Organism/group (*n*)	IMR	IPM	MEM	ATM	FEP	CAZ	TZP	LVX^a^	AMK^b^	CST^c^
NME										
Infection source^d^										
LRTI (973)	98.6	89.1	94.7	65.3	69.9	66.0	70.1	63.1	94.5	94.6
BSI (1021)	98.6	95.6	97.9	71.2	72.5	73.2	85.6	60.7	97.5	97.8
UTI (1140)	99.4	98.2	99.2	65.4	64.7	70.0	84.9	46.8	96.3	99.1
IAI (959)	98.3	95.0	97.2	71.7	76.3	74.0	78.5	65.7	96.7	98.2
Hospital ward^e^										
ICU (435)	98.4	91.3	95.9	64.8	69.2	67.1	74.0	63.4	95.9	94.0
Non-ICU (3645)	98.8	95.1	97.5	68.8	70.8	71.3	80.9	58.0	96.3	97.9
Length of hospitalization^f^										
<48 h (1881)	99.5	97.2	99.4	74.2	75.2	77.1	88.4	61.9	96.9	98.1
≥48 h (2211)	98.1	92.5	95.6	63.4	66.8	65.5	73.0	55.6	95.7	97.0
*P. aeruginosa*										
Infection source^d^										
LRTI (490)	86.5	63.5	72.7	66.9	78.0	74.9	70.4	59.0	93.5	100
BSI (72)	84.7	66.7	79.2	75.0	80.6	76.4	68.1	66.7	87.5	100
UTI (110)	64.5	50.9	60.9	63.6	59.1	58.2	55.5	43.6	66.4	97.3
IAI (65)	83.1	58.5	73.8	63.1	69.2	61.5	60.0	75.4	93.8	98.5
Hospital ward^e^										
ICU (146)	80.8	61.0	66.4	55.5	72.6	70.5	63.0	57.5	91.8	100
Non-ICU (588)	83.3	61.6	73.0	69.7	75.2	71.4	68.0	59.2	88.1	99.3
Length of hospitalization^f^										
<48 h (253)	88.5	68.4	77.9	73.1	78.7	79.4	75.5	60.1	91.7	100
≥48 h (483)	79.7	57.8	68.3	63.6	72.5	67.3	62.5	58.2	87.4	99.2

IMR, imipenem/relebactam; IPM, imipenem; MEM, meropenem; FEP, cefepime; CAZ, ceftazidime; TZP, piperacillin/tazobactam; LVX, levofloxacin; AMK, amikacin; CST, colistin; NME, non-Morganellaceae Enterobacterales; LRTI, lower respiratory tract infection; BSI, bloodstream infection; IAI, intra-abdominal infection.

^a^LVX data exclude *Salmonella* spp. (*n *= 6) as the MIC panel dilution range did not extend into susceptible concentrations for that genus.

^b^For AMK, percent susceptible values for NME were calculated using EUCAST 2023 breakpoints^4^ as the MIC panel dilution range did not cover the CLSI susceptible breakpoint. For *P. aeruginosa*, CLSI MIC breakpoints for AMK applicable to UTI isolates were applied to all isolates.^2^

^c^For CST, EUCAST 2023 MIC breakpoints^4^ were utilized because CLSI does not publish susceptibility breakpoints for Enterobacterales or *P. aeruginosa*.

^d^Seven NME isolates and 1 *P. aeruginosa* isolate did not have infection source specified.

^e^Twenty NME isolates and 4 *P. aeruginosa* isolates did not have hospital ward specified.

^f^Eight NME isolates and 2 *P. aeruginosa* isolates did not have length of hospitalization specified.

**Figure 1.**
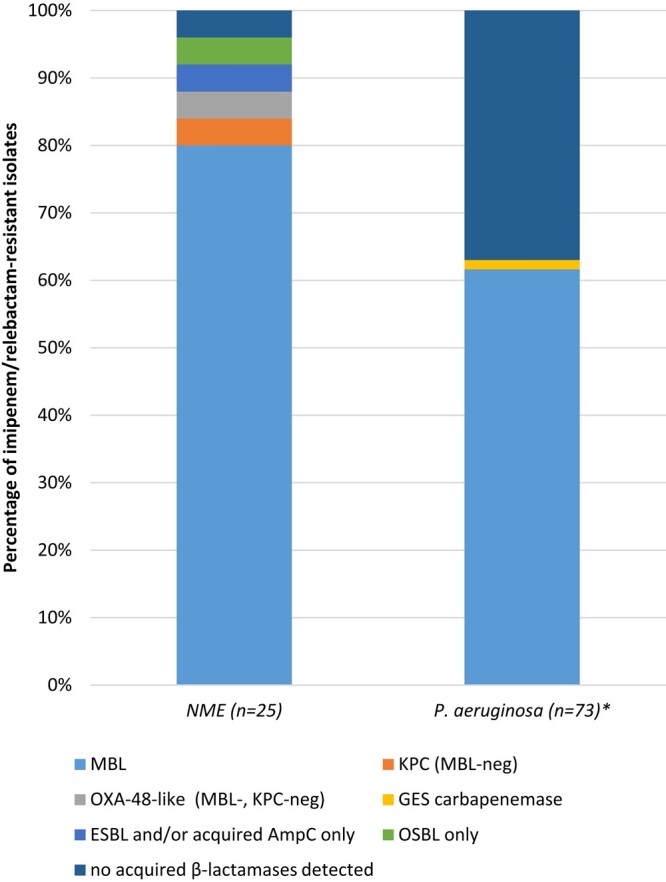
β-Lactamase carriage among characterized imipenem/relebactam-resistant NME (*n *= 25) and imipenem/relebactam-resistant *P. aeruginosa* (*n *= 73) from South Korea. *Thirteen imipenem/relebactam-resistant *P. aeruginosa* were not characterized for β-lactamase carriage.

Fifty-one isolates of NME met the criteria for molecular testing; 25 of the 51 isolates were imipenem/relebactam resistant (MIC ≥4 mg/L) and their results are shown in Figure 1. Eighty-four percent (21/25) of the imipenem/relebactam-resistant NME carried NDM (*n *= 20) or an OXA-48-like carbapenemase (*n *= 1), enzymes outside the inhibitory spectrum of relebactam. The remaining four isolates carried KPC (*n *= 1), SHV (*n *= 1), TEM (*n *= 1) or no acquired β-lactamase genes (*n *= 1). These four isolates included two *K. pneumoniae*, one *E. coli* and one *Klebsiella aerogenes* and presumably possess resistance mechanisms beyond β-lactamases that account for their imipenem/relebactam-resistant phenotype. One hundred and twenty-seven isolates of *P. aeruginosa* met the criteria for molecular testing; 86 of the 127 isolates were imipenem/relebactam resistant (MIC ≥8 mg/L). Thirteen of the 86 imipenem/relebactam-resistant *P. aeruginosa* isolates were not molecularly characterized. Among molecularly characterized imipenem/relebactam-resistant *P. aeruginosa*, 61.6% (45/73) harboured an MBL, including NDM (*n *= 30) and IMP (*n *= 15) enzymes. One isolate (1.4%) carried GES and for 27 isolates (37.0%) no acquired β-lactamases were observed, indicating the presence of other resistance mechanisms.

## Discussion

The prevalence of CRE and CRPA varies widely across countries in the Asia-Pacific region.^10–13^ In one previous publication, 98.8% of NME from patients in South Korea during 2017–20 were reported to be meropenem susceptible compared with meropenem percent susceptible values of 99.5%, 97.6%, 96.7%, 92.7%, 88.9% and 82.4% for isolates from patients in Hong Kong, Malaysia, Taiwan, the Philippines, Thailand and Vietnam, respectively.^10^ In the same publication, 72.1% of *P. aeruginosa* from patients in South Korea were reported to be meropenem susceptible compared with meropenem percent susceptible values of 86.2%, 83.7%, 81.4%, 81.0%, 67.1% and 55.3% for isolates from patients in Malaysia, Hong Kong, the Philippines, Taiwan, Thailand and Vietnam.^10^ In the current study, over a similar timeframe (2018–21), 97.3% of NME and 71.7% of *P. aeruginosa* from hospitalized patients in six sites in South Korea were meropenem susceptible (Table 1). Additional reports on clinical isolates of Gram-negative bacilli from South Korea include the 2015–19 ATLAS surveillance programme, which reported 17% carbapenem resistance and 7% ceftazidime/avibactam resistance among 609 isolates of *P. aeruginosa* collected in six hospital sites.^13^ Earlier, Kim and Park^14^ reviewed 2013–15 data from the Korean Antimicrobial Resistance Monitoring System (a network of 31 secondary and tertiary care hospitals) and reported <0.1% meropenem resistance in *E. coli*/year, 1% meropenem resistance in *K. pneumoniae*/year and 24%–27% meropenem resistance/year in *P. aeruginosa*. In 2012–15, the INFORM surveillance programme reported 99.7% of 1184 Enterobacterales isolates and 76.7% of 270 *P. aeruginosa* isolates from South Korea were meropenem susceptible.^12^ Cumulatively, these data suggest that in South Korea, carbapenem (meropenem) percent susceptible values among clinical isolates of Enterobacterales and *P. aeruginosa* may be decreasing.

DTR in patients with Gram-negative bloodstream infections is associated with higher mortality than those in other resistance categories such as MDR.^15^ Huh and colleagues^15^ studied 1167 episodes of monomicrobial Gram-negative bloodstream infections identified from the Korean Antimicrobial Resistance Surveillance Network in 2015–16 (14 hospital participants) and reported MDR and DTR rates for *E. coli* (MDR 12.3%; DTR 0%), *K. pneumoniae* (MDR 12.3%; DTR 1.4%) and *P. aeruginosa* (MDR 25.4%; DTR 9.0%). In the current study, which included aerobic or facultative Gram-negative isolates from intra-abdominal, urinary tract, lower respiratory tract and bloodstream infections combined, we observed that 24.1% and 2.3% of NME isolates were MDR and DTR, respectively, and 18.0% and 10.0% of *P. aeruginosa* isolates were MDR and DTR (Table 1). The 2012–15 INFORM surveillance programme reported lower rates of MDR for both Enterobacterales (16%; *n *= 1184) and *P. aeruginosa* (16%; *n *= 270) from South Korea^12^ than the current study, while the 2015–19 ATLAS programme reported more (22%) of *P. aeruginosa* to have an MDR phenotype.^13^ These percent differences between studies over time may be confounded by isolate source composition in each study.

In the Asia-Pacific region, in 2017–20, carbapenemase gene carriage rates in isolates of Enterobacterales and *P. aeruginosa* appeared highest in isolates from Vietnam (14.0% of isolates carried an MBL, 4.5% carried an OXA-48-like carbapenemase and 2.2% a KPC) while <1% of all NME from South Korea and Hong Kong carried a carbapenemase of any type.^10^ Data from the Korean Antimicrobial Resistance Monitoring System in 2015 showed that 48.3% of carbapenemases in Enterobacteriaceae were KPC, 26.4% were OXA-48, 16.6% were NDM, 6.5% were VIM, 1.6% were IMP and 0.5% were GES.^14^ In the current study, MBLs were commonly found (80%) in the few isolates of imipenem/relebactam-resistant NME identified (*n *= 25; 0.6% of all NME isolates were imipenem/relebactam resistant) and in 62% of molecularly characterized imipenem/relebactam-resistant *P. aeruginosa* [*n *= 73; 67% NDM, 33% IMP; 11.7% (86/738) of all *P. aeruginosa* isolates were imipenem/relebactam resistant] (Figure 1). Previously, the INFORM surveillance programme in 2015–17 reported that among 1226 Enterobacterales isolates collected at three sites in South Korea, <1% were carbapenemase positive, and among 307 *P. aeruginosa* isolates, 4.2% were carbapenemase positive (1% carbapenemase positive/MBL negative and 3.2% carbapenemase positive/MBL positive).^11^ The ATLAS surveillance programme from 2015–19 reported 17% of the carbapenem-resistant *P. aeruginosa* isolates carried a carbapenemase [77% were MBLs, of which IMP (85%) was most common].^13^

Relatively few published manuscripts have described the *in vitro* activity of imipenem/relebactam against clinical isolates of Gram-negative bacilli collected in South Korea.^10,16,17^ Most of these earlier publications grouped isolates from South Korea with isolates from other countries and reported collectively on isolates from the Asia-Pacific region.^16,17^ Only one previous publication has documented *in vitro* imipenem/relebactam susceptibility testing results specifically for clinical isolates of NME and *P. aeruginosa* from South Korea.^10^ The data presented in that publication were also from the SMART surveillance programme for an earlier 4 year time period (2017–20). The dataset in the current paper adds an additional year of data (2021), focuses on the most current SMART dataset available (2018 to 2021), and specifically details all MIC and demographic data available for isolates from South Korea, which was not available in the early publication.^10^ In the 2017–20 dataset, 99.0% of NME isolates (*n *= 3837) were imipenem/relebactam susceptible (current study: 98.8%), 98.8% were meropenem susceptible (current study: 97.3%) and 96.2% were imipenem susceptible (current study: 94.7%), while carbapenemase rates among in 2017–20 were estimated at 0.8% KPC (current study: 1.8%), 0.2% MBL (current study: 0.5%) and 0.1% OXA-48-like carbapenemases (current study: <0.1%).^10^ For *P. aeruginosa*, 86.1% of isolates (*n *= 707) in the 2017–20 dataset were imipenem/relebactam susceptible (current study: 82.8%), 72.1% were meropenem susceptible (current study: 71.7%) and 64.4% were imipenem susceptible (current study: 61.5%) while carbapenemase rates in 2017–20 were estimated at 4.7% MBL (current study: 6.5% MBL) and 0.3% GES carbapenemases (current study: 0.3% GES).^10^

Amikacin and colistin inhibited 96%–98% of NME and 89% to >99% of *P. aeruginosa* using EUCAST breakpoints or CLSI urine isolate breakpoints for amikacin tested against *P. aeruginosa* (Table 1). Even though these agents appear highly active *in vitro*, use of amikacin, other aminoglycosides and colistin is widely discouraged in clinical guidelines and by CLSI and EUCAST laboratory *in vitro* testing standards as both agents are strongly associated with major toxicity and therapeutic limitations.^2,4,15,18^

The data presented in this study followed regimented and consistent collection and testing processes; however, they are limited by the small annual sample size (250 Gram-negative isolates per medical centre per year) and the small number of participating medical centres per year (six). The data generated from isolates submitted by participating medical centres within South Korea should not be extrapolated to represent all isolates or geographical areas within that country.

We conclude that recent (2018–21) clinical isolates of Enterobacterales (99%) and *P. aeruginosa* (83%) collected in South Korea were highly susceptible to imipenem/relebactam. Based on *in vitro* data, imipenem/relebactam, if licensed in South Korea, may be a viable treatment option for many hospitalized patients infected with common Gram-negative pathogens including NME exhibiting MDR, DTR and carbapenem resistance and many β-lactam-resistant phenotypes of *P. aeruginosa*.

## Transparency declarations

M.G.W. and D.F.S. work for IHMA, which receives funding from Merck Sharp & Dohme LLC, a subsidiary of Merck & Co., Inc., Rahway, NJ, USA, for the SMART surveillance programme. J.A.K. is a consultant to IHMA. W.T.C., F.S., K.Y. and M.R.M. are employees of Merck Sharp & Dohme LLC, and own stock in Merck & Co., Inc., Rahway, NJ, USA. The IHMA authors and J.A.K. do not have personal financial interests in the sponsor of this manuscript (Merck Sharp & Dohme LLC). All authors provided analysis input and have read and approved the final manuscript.
